# Benign Paroxysmal Positional Vertigo After Mandibular Fractures

**DOI:** 10.7759/cureus.24442

**Published:** 2022-04-24

**Authors:** Khalid Bashir, Abdulla Yousuf, Amr Elmoheen

**Affiliations:** 1 Medicine, Qatar University, Doha, QAT; 2 Emergency Medicine, Hamad Medical Corporation, Doha, QAT; 3 Medical Education and Simulation, Hamad General Hospital, Doha, QAT

**Keywords:** ­trauma, emergency department maneuvers, bppv, oral and maxillofacial surgery, dizziness, mandibular fracture, benign paroxysmal positional vertigo

## Abstract

Benign paroxysmal position vertigo (BPPV) is a debilitating condition. BPPV is a peripheral vestibular disorder, and people with this condition experience varying levels of dizziness. BPPV, in most patients, is often overlooked as vertigo and dizziness may be triggered by an underlying chronic disease and disorder. Patients may be misdiagnosed or have delayed diagnosis, resulting in unnecessary health procedures. In this study, we present two cases of BPPV in a 29-year-old female and a 32-year-old male, who presented initially with fracture of the angle of left mandibles, which were treated surgically. Both patients developed BPPV secondary to head trauma leading to mandibular fracture. It is important to diagnose and treat BPPV early to prevent long-term disability.

## Introduction

Benign paroxysmal positional vertigo (BPPV) is a peripheral vestibular condition affecting at least 9% of adults aged between 18 and 35 years [1]. It is worth mentioning that BPPV sufferers experience a spinning sensation on certain head movements [2]. The etiology of BPPV remains unknown. BPPV is typically unilateral. In one study, 8% of patients developed BPPV after trauma [3].

It is important to note that some procedures such as preparation of implant beds, extraction of impacted wisdom teeth, procedures bordering around sinus floor elevation, and osteotomies done with the aid of surgical mallets and osteotomes can cause BPPV. These procedures transmit vibratory and percussive forces that can detach the otoliths, which results in them floating around in the endolymph resulting in BPPV [4]. It is important to diagnose and treat traumatic BPPV as the persistent rate, presence of disease in both ears, and recurrence rate are significantly higher in traumatic BPPV as compared to idiopathic BPPV [5].

Common postoperative complications associated with orthognathic surgery, sinus floor lift, and wisdom teeth extraction include nerve injuries, infection, poor fractures, acute sinusitis or chronic sinusitis, perforation of the Schneiderian membrane, and implant failure [6-8]. Postoperative BPPV is not a common disorder, and there are very few studies about this condition in the literature. But then, it should be considered by oral surgeons and maxillofacial surgeons.

We report two cases of BPPV in a 29-year-old female and a 32-year-old male, who presented with head trauma leading to the fracture of the angle of the right and left mandibles, which were treated surgically. A few days after discharge from the hospital, these patients presented to the emergency department with recurrent episodes of vertigo after changing positions in bed.

## Case presentation

Two patients aged 32 and 29 years initially presented with facial trauma leading to right and left mandibular fractures, respectively. After discharge from the hospital, these patients presented with recurrent episodes of vertigo after changing positions in the bed. Both patients were diagnosed with posterior canal BPPV after performing Dix-Hallpike maneuver and treated by particle repositioning maneuver with complete resolution of symptoms. 

The first patient was a 32-year-old, previously healthy, male who slipped and fell onto his right face. There was no loss of consciousness, vomiting, seizure, or weakness. On examination, in the emergency department, he had trismus and tender swelling on the right side of his mandible. There were no signs of the skull base or depressed skull fractures. The chest and abdomen examinations were unremarkable.

**Figure 1:**
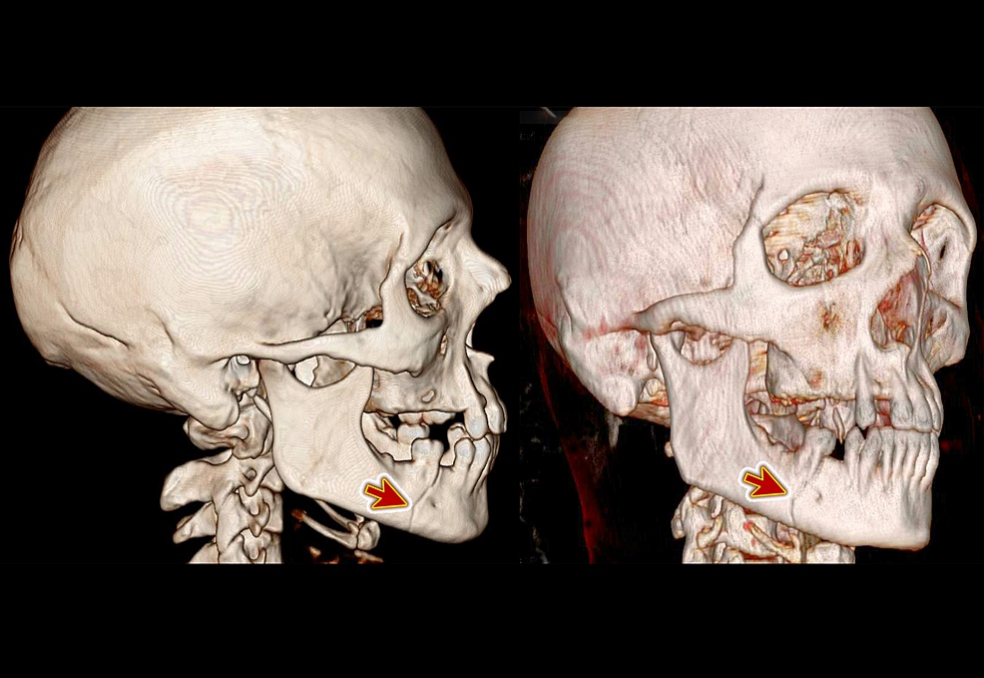
Three-dimensional computed tomography (3D CT) of the head and facial bone showing a fracture of the right side of the mandible (arrow)

The computed tomography (CT) of the facial bone showed a fracture of the right side of the mandible (Figure 1). The oro-maxillofacial surgeon admitted the patient for open reduction and internal fixation. The patient had an uneventful stay in the hospital and was then discharged home with no acute complications. He was followed up with the maxillofacial out-patient clinic.

Ten days after discharge, he presented with recurrent episodes of rotational vertigo after changing positions in the bed. He was diagnosed with posterior canal BPPV after performing Dix-Hallpike maneuver and treated by particle repositioning maneuver with complete resolution of symptoms.

The second patient was a 29-year-old woman, who was involved in a motor vehicle collision. She was the car driver, and the car slid and hit a wall while it was at a speed of 30 km/h. The patient was unrestrained, and the airbags did not deploy. After the accident, she presented to the emergency department with severe left-sided facial, neck, and right leg pain. She did not lose consciousness at the scene and had no other injuries. She was hemodynamically stable. She was conscious with no neurological deficit. There was swelling of the left side of the face, over the mandible and a step-deformity could be palpated. There were no signs of the skull base or depressed skull fractures. The chest and abdomen examinations were unremarkable.

**Figure 2:**
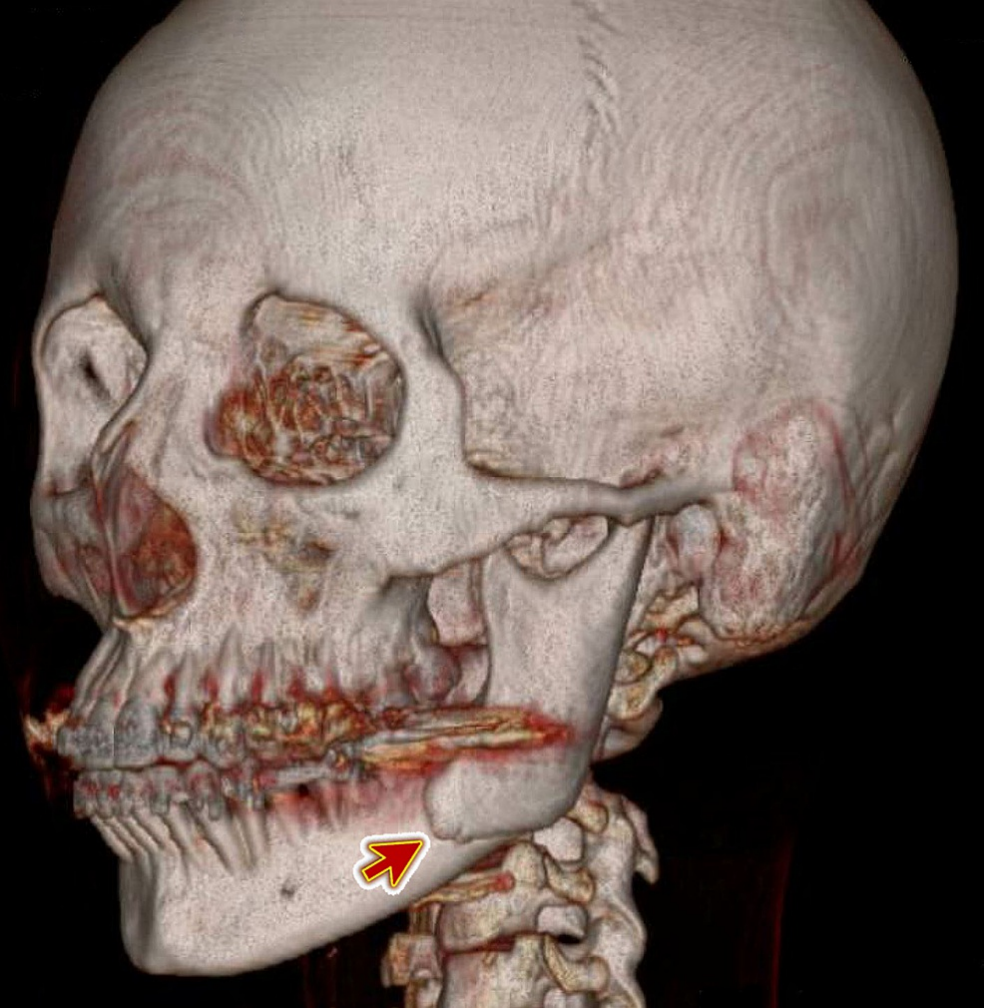
Three-dimensional computed tomography (3D CT) of the head and facial bone showing a fracture of the left side of the mandible (arrow)

The CT facial bone showed a fracture of the left side of the mandible (Figure 2). At her preference, she was admitted to the hospital by oro-maxillofacial surgeon managed conservatively. She was discharged home and followed-up in the outpatient clinic.

Seven days after discharge from the hospital, she had recurrent episodes of vertigo after changing positions in the bed. She presented to the emergency department with rotational vertigo, where she was diagnosed with posterior canal BPPV after performing Dix-Hallpike maneuver and treated by particle repositioning maneuver with complete resolution of symptoms.

## Discussion

Benign paroxysmal positional vertigo (BPPV) is a common pathological cause of dizziness. It has a purported prevalence of 11-64 per 100,000 people. It has a 2.4% lifetime prevalence [9,10]. It is important to note that BPPV can be correctly treated with the right canalith repositioning procedures (CRPs) [11]. BPPV is also a major cause of mental stress in patients, increases the individual’s balance, and jeopardizes the patient’s health [12-14].

BPPV occurs after the detachment of the otoconia from the otolithic organ into the semi-circular canal [15-18]. Although some experts attribute the otoconia detachment to degeneration of the otolith organ, the otoconia detachment has not been identified in most patients [19]. But only in some instances, the basis of the detachment from the otolithic organ is usually apparent. For instance, in both cases of this article, recurrent episodes were attributed to trauma which led to the fracture of the angle of the left mandibles. A fracture of the mandible results from trauma to the head; thus, the trauma caused by detachment and recurrent episodes of vertigo is relatively clear. Since the otolith organ is located in the temporal bone, it gets subjected to severe trauma during its fracture which may lead to otolith detachment leading to BPPV [20]. It is thought that BPPV caused by fracture of the temporal bone would damage more otolith organs than idiopathic BPPV. A 2021 study by Chang et al. suggests that BPPV secondary to trauma is more difficult to treat than idiopathic BPPV [21]. As such, we think that BPPV caused by temporal bone fracture would have greater resistance to CRPs than idiopathic BPPV. BPPV in head trauma has a 4.1-14.9% incident rate [22]. BPPV incidence is expected to be on the high side after fractures of the temporal bones.

It is important to note that the patient's surgical head position, hyperextended head, and duration of surgery with repeated vibratory and percussive stress all contribute to the otoconia's displacement into the posterior semi-circular canal, which starts off an abnormal signal that then causes vertigo when the patient sits. Postoperative BPPV features short-term recurrent vertigos accompanied by intense nystagmus. Risk factors for BPPV, such as high cholesterol levels, dyslipidemia, endocrinological disorders, vascular problems, belonging to the female gender, perimenopausal age (50-60 years), vascular problems, migraine, cranial trauma, autoimmune disease, neurologic disorders, hypovitaminosis, and inner ear flogosis, may enhance the onset of postoperative vertigo [23].

It is imperative that emergency physicians are trained to diagnose and treat BPPV in patients as early as possible to prevent disability [24-26]. As far as we are aware, no previous cases of head trauma and fractured mandible leading to BPPV have been reported. Head trauma caused both the mandible fracture and the BPPV. Head trauma is a known cause of BPPV. This is a case of the two effects of head trauma occurring. The case series stresses the importance of managing a possible remediable medical disorder that can develop after a head trauma together.

## Conclusions

Head trauma caused both the mandible fracture and the BPPV. Head trauma is a known cause of BPPV. This is a case of the two effects of head trauma occurring together. If BPPV is treated early by appropriately trained physicians, it will prevent long-term disability caused by recurrent episodes of vertigo.
